# Supplementary material for: Chromothripsis during telomere crisis is independent of NHEJ, and consistent with a replicative origin
Source: Genome Res. 2019 May;29(5):737–49. doi: 10.1101/gr.240705.118 (PMC6499312; doi:10.1101/gr.240705.118)
Supplement: Supplemental Material [file supp_gr.240705.118_Supplemental_file_1.zip › contigs/annotated_contigs/DB49/contig.2.DB49_length_427_mean_cov_7.74004683841.docx]

**DB49_length_427_mean_cov_7.74004683841**

```
TTAAACAATAAATGTCATGGCTGGACGCAGTGGCTCATGCCTGTA|ATCCCAGCACTTTGGGAGGCCGAGGTGGGAGGATCAC|GAGGT
 >chr20:48738939-48739021 + E=9e-26 p=0e+00   >chr16:32449610-32449989 + E=1e-183
CAGGAGATCGAGACCATCCTGGCTAACACGGTGAAACTCTGTCTCTACTAAAAATACAAAAAACTAGCCAGGCATGGTGGTAGGTGTCT

GTAGTCCCGGCTACTCGGGAGGCTGAGGCAGAAGAATCACTTGAACCTGGGAGGCAGAGGTTGCAGTGAGCCAAGATCACACCACTGCA

CTCCAGCCTGGGTGACAGAGTGAGACTCCATCTCAAAAAATAAAATAAAATAAAATAAAATAAAAAACAAATAAATACAAGATTGTTGT

TTCTTATAAACTTTTTTTGTATCTTTGCCTATTTTTTTCACTGTCTAAGGAATTTTTATTAAAGCAAAAT|GTT
```
